# Fetal exposure markers of dioxins and dioxin-like PCBs

**DOI:** 10.1007/s11356-018-1447-y

**Published:** 2018-02-15

**Authors:** Erik Lampa, Akifumi Eguchi, Emiko Todaka, Chisato Mori

**Affiliations:** 1 UCR - Uppsala Clinical Research Center, Uppsala Science Park, Hubben, 751 85 Uppsala, Sweden; 2 Center for Preventive Medical Sciences, Chiba University, Chiba, Japan

**Keywords:** PCB, Dioxin, Fetal exposure, Multivariate analysis

## Abstract

Fetal exposure to polychlorinated biphenyls (PCBs), polychlorinated-*p*-dibenzodioxins (PCDDs), and polychlorinated dibenzofurans (PCDFs) have been associated with a number of adverse health outcomes. Although the placenta acts as a barrier between the mother and the fetus, these contaminants transfer through the placenta exposing the fetus. Several studies have investigated placental transfer, but few have assessed the co-variation among these contaminants. Maternal blood, cord blood, and cord tissue were collected from 41 Japanese mother-infant pairs and analyzed for dioxin-like PCBs and PCDD/Fs. Hierarchical cluster analysis followed by principal component analysis were used to assess the co-variation. Two stable clusters of dioxin-like PCBs were found in maternal and cord blood. One cluster of low/medium chlorinated dioxin-like PCBs was present in all three matrices with 2,3’,4,4’,5-PeCB(#118) and 3,3’,4,4’,5-PeCB(#126) explaining the majority of the clusters’ variances. Medium/high chlorinated dioxin-like PCBs clustered in maternal blood and cord blood but not in cord tissue. 2,3,4,4’,5-PeCB(#114) and 2,3,3’,4,4’,5,5’-HpCB(#189) explained the majority of the clusters’ variances. There was a substantial correlation between the sum of dioxin-like PCBs and total PCDD/F in all three matrices. The sum of the four suggested PCBs plus 3,3’,4,4’-TeCB(#77) correlated well with total PCDD/F in all three matrices. Apart from the dioxin-like PCBs, little co-variation existed among the studied contaminants. The five PCBs can be used as fetal exposure markers for dioxin and dioxin-like PCBs in maternal and cord blood respectively. In cord tissue, more higher chlorinated dioxin-like PCBs need to be measured as well.

**Electronic supplementary material:**

The online version of this article (10.1007/s11356-018-1447-y) contains supplementary material, which is available to authorized users.

## Introduction

Polychlorinated biphenyls (PCBs), polychlorinated-*p*-dibenzodioxins (PCDDs), and polychlorinated dibenzofurans (PCDFs) are widespread environmental contaminants that have been associated with numerous adverse health effects in humans such as increased cancer risk (Kogevinas 2001; Oakley et al. 1996), reproductive disorders (Crain et al. 2008), and nervous system damage (Grandjean and Landrigan 2006). Although the use and production of them is banned in large parts of the world, they continue to persist in the environment and accumulate in human adipose tissue. Of special concern is the exposure of a developing fetus because of potentially increased vulnerability during critical development stages. Epidemiological studies have linked maternal exposure to PCBs and PCDD/Fs to health outcomes such as growth restriction (Govarts et al. 2012), neurobehavioral deficits (Grandjean et al. 2001), and impaired immune response in the child (Svensson et al. 1994). Accumulated contaminants are passed from the mother to the fetus through the placenta and the umbilical cord. Although the placenta acts as a barrier protecting the growing fetus, PCBs and dioxins as groups easily pass the placenta (Lancz et al. 2014). However, PCBs and PCDD/Fs are mixtures of different congeners, each with their own physiochemical properties. Congeners with lower molecular weight are more likely to pass the placenta than congeners with higher molecular weight (Eguchi et al. 2015; Mori et al. 2014). While placental transfer has been investigated in several studies, few studies have investigated the co-variation of PCBs and dioxins. The aim of this study was to assess the degree of co-variation among dioxins and dioxin-like PCBs in maternal blood, cord blood, and cord tissue and to find suitable markers of exposure.

## Material and Methods

### Data collection

Maternal blood, umbilical cord blood, and cord tissue were collected from 41 mother-infant pairs at Chiba University Hospital and at various other obstetrics units in Japan during 2003 and 2004. Blood samples were stored at − 20 °C until use. Umbilical cords were collected immediately after delivery and stored in acetone-washed glass bottles at − 20 °C until use. Concentrations of seven PCDDs, 10 PCDFs and 12 dioxin-like PCBs were measured in all three matrices. TEQ-calculated values, using the WHO 2005 Toxic Equivalence Factors (TEF) (Van den Berg et al. 2006), were used in all statistical analyses. Further details on the data collection and chemical analyses can be found in Sakurai et al. (2004), Kawashiro et al. (2008), and Mori et al. (2014). The age distribution of the mothers is given in Table 1. The study was approved by the Congress of Medical Bioethics at Chiba University, and all samples were obtained after receipt of written informed consent.

**Table 1:** Age distribution of the 41 mothers participating in the study

Age group (years)	*N*
20–24	2
25–29	11
30–34	20
35 and above	8
Total	41

### Statistical methods

Hierarchical cluster analysis, which groups the contaminants into distinct clusters based on a measure of similarity between them, was used to quantify how the contaminants co-vary within each data set. Here, 1 − |*ρ*|, where *ρ* is Spearman’s rank correlation coefficient and |.| denotes the absolute value, was used as the dissimilarity measure. Cluster stability was evaluated using multistage multiscale bootstrap (Shimodiara 2005) in which the clustering in repeated resamples from the data, with sampling fractions ranging from 0.5 to 1.4, is compared to the observed clustering. The agreement between observed clustering and resampled clustering is called the bootstrap probability (BP) value, and *z*-values for the BP-values for each sampling fraction were calculated according to *z* = Φ^− 1^(BP) where Φ^− 1^(⋅) is the inverse of the standard normal distribution function. For each cluster, a line is then fitted to the *z*-values by $z(\lambda )=\alpha \sqrt {\lambda }+\beta \sqrt {\lambda ^{-1}}$ where *λ* denotes the sampling fraction. Asymptotically unbiased probability values (AU *p* values) were then calculated for each cluster by AU = Φ(−*α* + *β*). Stable clusters were defined as clusters with AU ≥ 0.95.

To assess whether a smaller subset of contaminants could explain the majority of the variability within each identified cluster, we performed a principal component analysis within each stable cluster containing more than two contaminants. The contaminants were first transformed to zero mean and unit variance by the maximum generalized variance (MGV) method (Harrell 2001). The MGV algorithm transforms the contaminants in an iterative fashion where each contaminant is represented using restricted cubic splines. In each step, canonical variate analysis is used to find optimal transformations so that each contaminant is most similar to predictions from the other contaminants. The process is repeated until the transformations converge. Each stable cluster was represented by the first principal component score. We then used a multiple linear regression model to predict the score, using restricted cubic splines with knots placed at the 10^*th*^, 50^*th*^, and 90^*th*^ percentiles of each contaminant’s distribution for each constituent contaminant, and measured the decrease in the coefficient of determination, *R*^2^, from stepwise deletions until *R*^2^ ≈  0.95. The contaminants left in the model were deemed suitable markers for that cluster.

Associations between ΣPCB_R_ and ΣPCDD/F, where PCB_R_ is the subset of dioxin-like PCBs needed to explain most of the variance in all dioxin-like PCBs, in maternal blood, cord blood and cord tissue were quantified using Spearman’s rank correlation, *ρ*. Ninety-five percent confidence intervals for *ρ* were calculated using the bootstrap with 10,000 replicates. We also estimated the correlation of the individual contaminants between the three matrices. Contaminant values below the limit of detection (LOD) were set to LOD/2. All analyses were done using R version 3.1.0 (R Core Team 2014) with the pvclust (Suzuki and Shimodaira 2011) and Hmisc (Harrell 2014) packages.

## Results

Table 2 shows descriptive statistics for the data. Following Tsukimori et al. (2013), only compounds with ≥ 50% of the samples above the LOD in all three matrices were considered for further statistical analysis.

**Table 2:** Descriptive statistics presented as median (IQR)

	Maternal blood		Cord blood		Cord tissue	
Contaminant	*N*	Median (IQR)	*N*	Median (IQR)	*N*	Median (IQR)
2,3,7,8-TeCDD	34	0.76 (0.66–0.96)	4	1.0 (0.96–1.0)	14	0.83 (0.73–1.1)
1,2,3,7,8-PeCDD	41	3.7 (2.7–4.3)	36	2.0 (1.6–2.5)	40	3.8 (2.9–5.0)
1,2,3,4,7,8-HxCDD	39	0.16 (0.12–0.2)	4	0.15 (0.14–0.17)	15	0.15 (0.12–0.16)
1,2,3,6,7,8-HxCDD	41	1.5 (1.1–1.8)	41	0.68 (0.56–0.8)	41	0.94 (0.67–1.1)
1,2,3,7,8,9-HxCDD	41	0.23 (0.18–0.3)	32	0.18 (0.14–0.21)	24	0.23 (0.17–0.3)
1,2,3,4,6,7,8-HpCDD	41	0.11 (8.7e-2–0.16)	41	4.6e-2 (3.5e-2–6.8e-2)	41	6.3e-2 (5.2e-2–8.5e-2)
OCDD	41	1.6e-2 (1.2e-2–2.3e-2)	41	4.1e-3 (2.7e-3–5.8e-3)	41	5e-3 (4.3e-3–7.5e-3)
2,3,7,8-TeCDF	40	7.8e-2 (6.6e-2–9.4e-2)	24	7.3e-2 (6.4e-2–8.9e-2)	15	7.8e-2 (5.1e-2–0.11)
1,2,3,7,8-PeCDF	21	2.0e-2 (1.7e-2–2.4e-2)	5	2.8e-2 (2.7e-2–3.2e-2)	6	0.02 (1.8e-2–2.7e-2)
2,3,4,7,8-PeCDF	41	3.1 (2.6–4.2)	41	1.5 (1.2–2)	41	3.1 (2.5–3.9)
1,2,3,4,7,8-HxCDF	41	0.25 (0.21–0.33)	39	0.14 (0.11–0.18)	40	0.27 (0.22–0.31)
1,2,3,6,7,8-HxCDF	41	0.33 (0.29–0.45)	41	0.23 (0.18–0.3)	41	0.31 (0.24–0.36)
1,2,3,7,8,9-HxCDF	0	–	0	–	0	–
2,3,4,6,7,8-HxCDF	39	0.14 (9.8e-2–0.18)	16	0.11 (0.1–0.12)	29	0.12 (8.9e-2–0.16)
1,2,3,4,6,7,8-HpCDF	41	2.3e-2 (1.9e-2–3.4e-2)	39	1.7e-2 (1.4e-2–2.4e-2)	39	2.4e-2 (1.8e-2–2.8e-2)
1,2,3,4,7,8,9-HpCDF	0	–	0	–	0	–
OCDF	3	1.4e-4 (1.3e-4–1.4e-4)	0	–	0	–
$\boldsymbol {\sum } {PCDD/F}$		10 (8.2–13)		4.8 (3.5–5.1)		9.6 (7.0–12)
3,3’,4,4’-TeCB(#77)	41	5.3e-4 (4.2e-4–6.6e-4)	40	3.8e-4 (3.1e-4–5.2e-4)	40	3.8e-4 (3.1e-4–5.3e-4)
3,4,4’,5-TeCB(#81)	41	1.1e-4 (8.5e-5–1.6e-4)	18	9.2e-5 (7.6e-5–1.1e-4)	33	1.3e-4 (9.4e-5–1.7e-4)
2,3,3’,4,4’-PeCB(#105)	41	0.1 (7.1e-2–0.15)	41	4.9e-2 (3.1e-2–6.8e-2)	41	0.075 (0.06–0.11)
2,3,4,4’,5-PeCB(#114)	41	0.12 (9.5e-2–0.2)	41	5.8e-2 (4.0e-2–9.0e-2)	41	9.2e-2 (7.9e-2–0.13)
2,3’,4,4’,5-PeCB(#118)	41	0.48 (0.31–0.64)	41	0.21 (0.14–0.33)	41	0.35 (0.24–0.44)
2’,3,4,4’,5-PeCB(#123)	41	6.9e-3 (5.2e-3–9.3e-3)	41	3.4e-3 (2.7e-3–5.8e-3)	41	5.4e-3 (3.8e-3–7.2e-3)
3,3’,4,4’,5-PeCB(#126)	41	2.5 (1.8–3.3)	41	1 (0.76–1.5)	41	1.7 (1.3–2.3)
2,3,3’,4,4’,5-HxCB(#156)	41	0.81 (0.58–1.2)	41	0.27 (0.2–0.44)	41	0.46 (0.39–0.62)
2,3,3’,4,4’,5’-HxCB(#157)	41	0.2 (0.15–0.3)	41	0.08 (5.9e-2–0.13)	41	0.12 (0.10–0.17)
2,3’,4,4’,5,5’-HxCB(#167)	41	6e-3 (4.5e-3–8.2e-3)	41	2.3e-3 (1.8e-3–3.5e-3)	41	3.6e-3 (2.6e-3–4.7e-3)
3,3’,4,4’,5,5’-HxCB(#169)	41	0.22 (0.18–0.29)	41	7.6e-2 (5.8e-2–0.10)	41	0.12 (9.8e-2–0.15)
2,3,3’,4,4’,5,5’-HpCB(#189)	41	1.8e-2 (1.4e-2–2.4e-2)	41	5.1e-3 (4.0e-3–7.6e-3)	41	8.1e-3 (6.6e-3–1.0e-2)
$\boldsymbol {\sum } {PCB}$		4.6 (3.3–6.0)		1.8 (1.2–2.5)		3.0 (2.2–3.7)
$\boldsymbol {\sum } {PCDD/F~and~PCB}$		15 (12–19)		6.8 (4.6–8.1)		13 (9.6–16)

*N* is the number of observations above the LOD. *x* e±*y* means *x* ⋅ 10^±*y*^. All units are pg-TEQ/g-fat

**Fig. 1:**
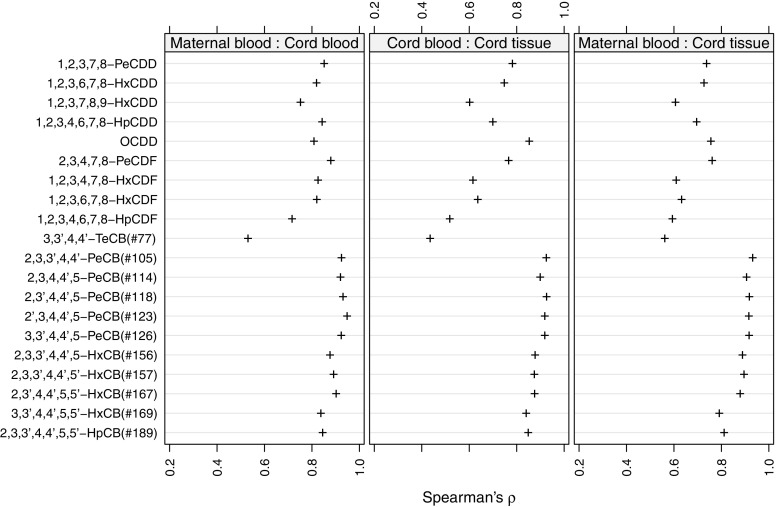
Spearman’s rank correlation between individual PCBs and PCDD/Fs in all three matrices

Figure 1 shows the correlation coefficients for the dioxin-like PCBs and PCDD/Fs between all three matrices. Correlations between the dioxin-like PCBs in the different matrices were generally high with the exception of 3,3’,4,4’-TeCB(#77). The PCDD/Fs correlated well between maternal and cord blood, but the correlation dropped somewhat between the other matrices.

**Fig. 2:**
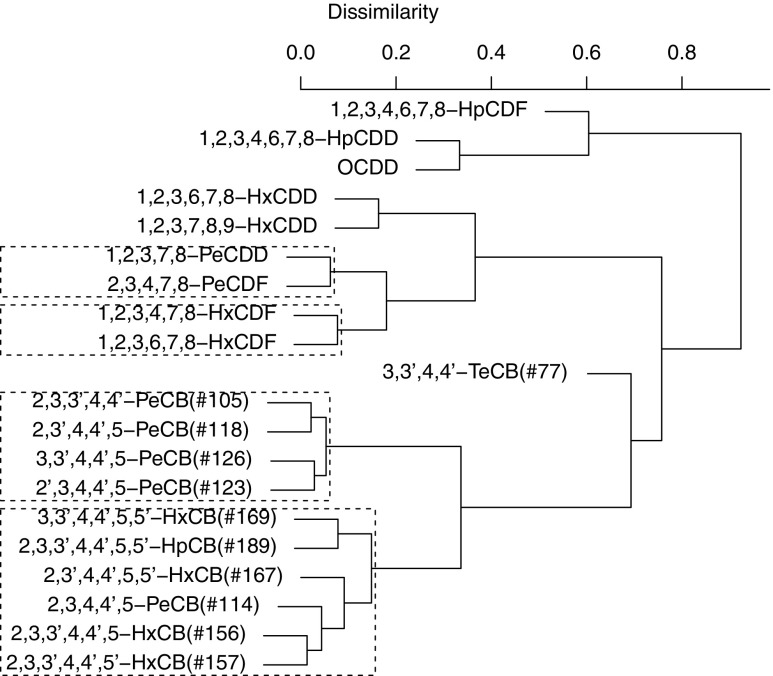
Dendrogram showing the results of the cluster analysis applied in the maternal blood data. Stable clusters are enclosed in dashed boxes

**Fig. 3:**
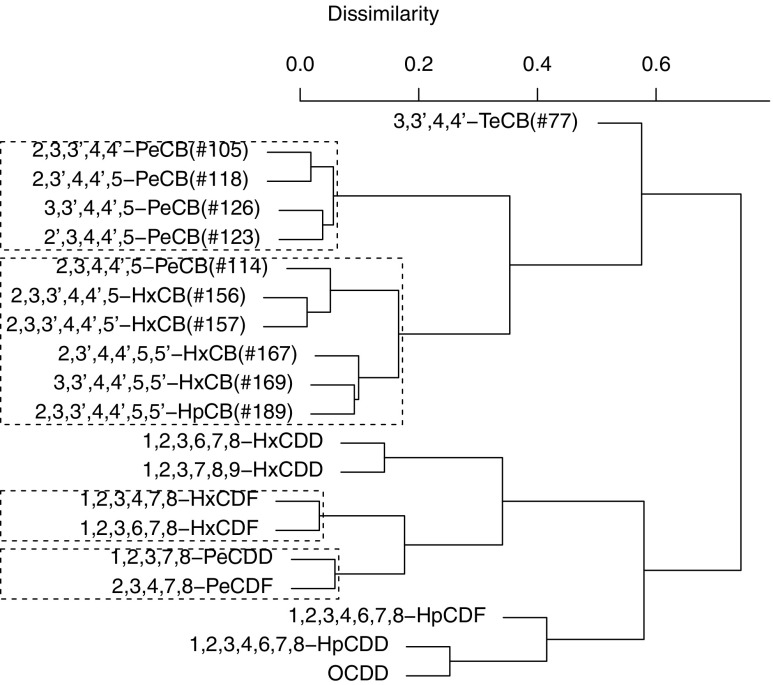
Dendrogram showing the results of the cluster analysis applied in the cord blood data. Stable clusters are enclosed in dashed boxes

**Fig. 4:**
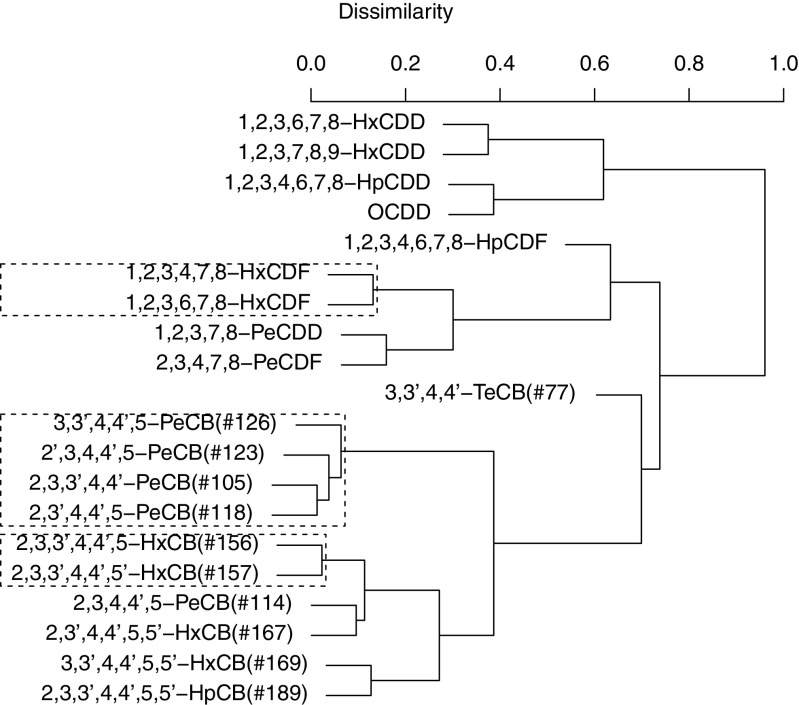
Dendrogram showing the results of the cluster analysis applied in the cord tissue data. Stable clusters are enclosed in dashed boxes

Dendrograms from the hierarchical cluster analyses are shown in Figs. 2, 3, and 4. The *y*-axis in each figure represents the dissimilarity between the contaminants, with higher values reflecting less correlated contaminants. Stable clusters as judged by the bootstrap procedure are enclosed in dashed boxes.

### Stable clusters

One stable cluster consisting of the low/medium chlorinated PCBs 2,3,3’,4,4’-PeCB(#105), 2,3’,4,4’,5-PeCB(#118), 2’,3,4,4’,5-PeCB(#123), and 3,3’,4,4’,5-PeCB(#126) was present in all matrices. In addition, a stable cluster consisting of the medium/high chlorinated PCBs 2,3,3’,4,4’,5-HxCB(#156), 2,3,3’,4,4’,5’-HxCB(#157), 2,3’,4,4’,5,5’-HxCB(#167), 3,3’,4,4’,5,5’-HxCB(#169), 2,3,3’,4,4’,5,5’-HpCB(#189) as well as 2,3,4,4’,5-PeCB(#114) was found in maternal blood and cord blood. The cluster could also be seen in cord tissue; although, only a small sub-cluster consisting of 2,3,3’,4,4’,5-HxCB(#156) and 2,3,3’,4,4’,5’-HxCB(#157) was deemed stable. Although 3,3’,4,4’-TeCB(#77) seemed to cluster with the other dioxin-like PCBs, it was not part of any stable cluster. No cluster with more than two constituent PCDD/Fs was seen in any of the matrices; although, a small cluster consisting of 1,2,3,4,7,8-HxCDF and 1,2,3,6,7,8-HxCDF was deemed stable in all three matrices. Both 1,2,3,7,8-PeCDD and 2,3,4,7,8-PeCDF clustered in maternal blood and cord blood.

### Suitable markers

Table 3 shows the percent variance explained by the first principal component as well as *R*^2^ from the regression models and the constituent contaminants. Generally, the first principal component explained a large amount of the variance in each cluster suggesting strong correlations among the constituent contaminants. For the low/medium chlorinated cluster, 2,3’,4,4’,5-PeCB(#118) and 3,3’,4,4’,5-PeCB(#126) could be seen as suitable markers in all three matrices. For the medium/high chlorinated cluster, 2,3,4,4’,5-PeCB(#114) and 2,3,3’,4,4’,5,5’-HpCB(#189) could be suitable markers in maternal blood and cord blood.

**Table 3:** Explained variance of the first principal component ($R_{\text {PC1}}^{2}$), *R*^2^ and constituent contaminants for a model predicting the first principal component

Matrix	Cluster	$R_{\text {PC1}}^{2}$	*R* ^2^	PCB_R_
Maternal blood	Low/medium chlorinated	0.97	1.00	2,3’,4,4’,5-PeCB(#118)
				3,3’,4,4’,5-PeCB(#126)
	Medium/high chlorinated	0.94	0.99	2,3,4,4’,5-PeCB(#114)
				2,3,3’,4,4’,5,5’-HpCB(#189)
Cord blood	Low/medium chlorinated	0.94	1.00	2,3’,4,4’,5-PeCB(#118)
				3,3’,4,4’,5-PeCB(#126)
	Medium/high chlorinated	0.97	0.99	2,3,4,4’,5-PeCB(#114)
				2,3,3’,4,4’,5,5’-HpCB(#189)
Cord tissue	Low/medium chlorinated	0.96	0.99	2,3’,4,4’,5-PeCB(#118)
				3,3’,4,4’,5-PeCB(#126)

### Relationship between ΣPCB_R_ and ΣPCDD/F

**Fig. 5:**
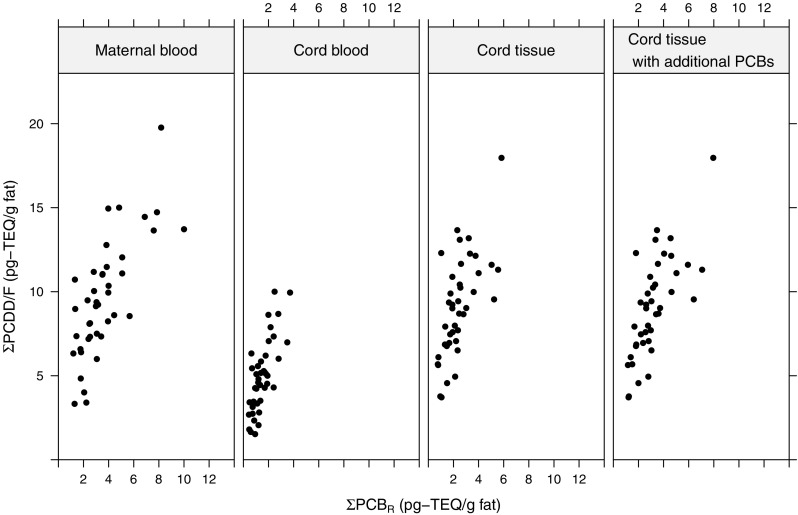
Associations between total PCDD/F and PCB_R_ in the three matrices. The rightmost panel includes additional higher chlorinated dioxin-like PCBs in the sum

The relationships between ΣPCB_R_ and ΣPCDD/F in the three matrices can be seen in Fig. 5 and Table 4. The medium/high chlorinated PCBs 2,3,4,4’,5-PeCB(#114) and 2,3,3’,4,4’,5,5’-HpCB(#189) are part of PCB_R_ in cord tissue despite the lack of a larger stable medium/high chlorinated cluster. Figure 5 contains an extra panel for cord tissue. In this panel, PCB_R_ consists of 3,3’,4,4’-TeCB(#77), 2,3’,4,4’,5-PeCB(#118), and 3,3’,4,4’,5-PeCB(#126) as well as all the dioxin-like PCBs in the medium/high chlorinated cluster. Table 4 shows the values of Spearman’s *ρ* and the 95% nonparametric bootstrap confidence intervals. ΣPCB_R_ and ΣPCDD/F were positively correlated in all three matrices with *ρ* being highest in maternal blood followed by cord blood and cord tissue; although, the differences were small. The correlation between ΣPCB_R_ and ΣPCDD/F in cord tissue, when additional higher chlorinated dioxin-like PCBs were added to the sum of dioxin-like PCBs, was higher than the correlation when the additional dioxin-like PCBs were not added to the sum.

**Table 4:** Spearman’s rank correlation and nonparametric bootstrap confidence intervals between the sum of 3,3’,4,4’-TeCB(#77) and the representative dioxin-like PCBs and total PCDD/F

	Spearman’s *ρ*	95% CI
Maternal blood	0.74	0.53–0.86
Cord blood	0.73	0.46–0.86
Cord tissue	0.68	0.39–0.82
Cord tissue^a^	0.73	0.46–0.85

^a^ With additional higher chlorinated dioxin-like PCBs

## Discussion

This study has investigated the co-variation among PCDD/Fs and dioxin-like PCBs in three different matrices: maternal blood, umbilical cord blood, and umbilical cord tissue. We observed one stable cluster containing four PentaCBs in all three matrices (Figs. 2, 3, and 4) suggesting that these PCBs transfer through the placenta and accumulate in the cord tissue in a similar manner. Based on our results, measuring 3,3’,4,4’,5-PeCB(#126) in addition to 2,3’,4,4’,5-PeCB(#118) would be enough to get a good picture of fetal exposure to these PCBs. Furthermore, 3,3’,4,4’,5-PeCB(#126) has the strongest toxicity among the co-planar PCBs and can, from a toxicity viewpoint, be used as a marker PCB. Since the correlations between the PCBs in different matrices were high (see Fig. 1), a sample from maternal blood could be used as a proxy for fetal exposure to dioxin-like PCBs and PCDD/Fs. For a biomonitoring study however, it is necessary to measure the contaminants in umbilical cord tissue as levels vary between the matrices (Fukata et al. 2005). Samples taken in maternal blood and cord blood also represent exposure levels in mothers while samples taken in cord tissue represent the cumulative exposure during the gestation period.

Another PCB cluster consisting mainly of HexaCBs, and one PentaCB, was observed in maternal blood and cord blood but not in cord tissue. Placental transfer is mainly determined by the molecular weight, with lighter compounds transferring more easily than heavier ones (Eguchi et al. 2015; Lancz et al. 2014). Although higher chlorinated PCBs are heavier than lower chlorinated ones, the weight difference is not enough to dissolve the cluster in cord blood. One reason for the lack of the highly chlorinated PCB cluster in cord tissue could be the lower levels of highly chlorinated PCBs in cord tissue. Lower levels, close to the LOD, have a high relative uncertainty, and although a rank-based measure was used in the clustering, it is possible that this uncertainty in measured levels has contributed to the uncertainty in the cluster. The two PCBs present in the small stable cluster in cord tissue are present in higher levels than the other PCBs included in the corresponding cluster in maternal blood or cord blood. The human body metabolizes PCBs differently depending on the degree of chlorination, with lower chlorinated PCBs generally being metabolized quicker than higher chlorinated PCBs (Grimm et al. 2015). This difference in metabolism could also explain the presence of a cluster of lower/medium chlorinated PCBs in cord tissue.

Clusters similar to what we observed in maternal blood were observed in data from the National Health and Nutrition Survey (NHANES) and in an elderly population in Sweden (Lampa et al. 2012) where the degree of chlorination in part explained the clustering. It is obvious from our results that the degree of chlorination is a major determinant of the clustering in our data; although, the PentaCBs did not cluster together with 2,3,4,4’,5-PeCB(#114) which appeared in the medium/higher chlorinated cluster and especially clustered with 2,3,3’,4,4’,5-HxCB(#156) and 2,3,3’,4,4’,5’-HxCB(#157). The physiochemical properties of 2,3,4,4’,5-PeCB(#114) is more similar to those of 2,3’,4,4’,5-PeCB(#118) than those of 2,3,3’,4,4’,5-HxCB(#156) and 2,3,3’,4,4’,5’-HxCB(#157) (Eguchi et al. 2015), so different exposure sources could explain why 2,3,4,4’,5-PeCB(#114) did not cluster with the other PentaCBs. Alcock et al. (1998) report that Aroclor formulations have dominated exposure to the lower chlorinated PCBs, especially 3,3’,4,4’-TeCB(#77), while combustion processes were found to be the source of higher chlorinated PCBs. It is possible that exposure to 2,3,4,4’,5-PeCB(#114) follows similar routes as exposure to higher chlorinated PCBs; although, we have found no literature sources confirming this. A similar pattern was found in 41 Yucheng victims (Hsu et al. 2005) in which 2,3,4,4’,5-PeCB(#114), 2,3,3’,4,4’,5-HxCB(#156), and 2,3,3’,4,4’,5’-HxCB(#157) were found to co-vary. Although 2,3’,4,4’,5-PeCB(#118) was measured, the number of nondetectable values was high for that congener and it was not included in the principal component analysis. Hsu et al. (2005) also studied PCDD/Fs and found similar principal component loadings for 1,2,3,4,7,8-HxCDF and 1,2,3,6,7,8-HxCDF which also clustered together in our data.

The population from which the individuals in the present study is sampled is a very homogeneous one, and the fact that similar clustering patters are observed in different populations of different ages add strength to our findings. The sum of the representative PCBs and total PCDD/F correlated well in all three matrices; although, the correlation was slightly lower in cord blood and cord tissue than in maternal blood (see Table 4). Levels of total dioxin-like PCB and PCDD/F decreased in cord blood compared to maternal blood but rose again in cord tissue (see Table 2) which fits well with previous results regarding levels and correlations of PCBs and PCDD/Fs in the three matrices (Fukata et al. 2005; Grandjean et al. 2001; Waliszewski et al. 2000; Walker et al. 2003; Sala et al. 2001).

Based on the results in this study, the sum of 3,3’,4,4’-TeCB(#77), 2,3’,4,4’,5-PeCB(#118), 3,3’,4,4’,5-PeCB(#126), 2,3,3’,4,4’,5,5’-HpCB(#189), and 2,3,4,4’,5-PeCB(#114) could well be used as a proxy for fetal exposure to total dioxin-like PCB and PCDD/F in studies taking a whole mixtures approach. For measurements made in cord tissue, we suggest that additional higher chlorinated dioxin-like PCBs should be measured and included in the sum.

Two major limitations of this study are the low sample size and the large number of PCDD/Fs below the LOD. It is therefore important that these results are replicated in larger studies in different populations. The population studied cannot be considered highly exposed to PCBs and PCDD/Fs when compared to those exposed by, e.g., the Seveso incident or Agent Orange in Vietnam. However, Alcock et al. (1998) reported that the contribution of dioxin-like PCBs to total TEQ was comparable to that of PCDD/Fs in normal populations and in human milk fat and is usually dominated by PCB 156 (23–36%), PCB 126 (22–37%), and PCB 118 (10–17%), which collectively constitute between 50 and 90% of the ΣPCB-TEQ.

The number of measurements below the LOD was high for some PCDD/Fs, and those were not included in the final analysis. Values below the LOD were imputed by LOD/2 resulting in observations below the LOD being given the same value. The rank-based measure splits tied values randomly when assigning ranks and could thus dilute any relationship. This can also have contributed to the lack of stable clusters among the PCDD/Fs. This study focused on dioxin-like PCBs, and data for nondioxin-like PCBs was not available. It would be valuable to repeat these analyses in data where both dioxin-like and nondioxin-like PCBs were measured to assess if any nondioxin-like PCBs could be used as fetal exposure markers.

In conclusion, there was relatively little co-variation between the studied contaminants suggesting that to get a complete picture of fetal exposure, several contaminants need to be measured. Five dioxin-like PCBs can be used as representative PCBs for total fetal dioxin-like PCB exposure. Our results also suggest that the sum of the representative dioxin-like PCBs and 3,3’,4,4’-TeCB(#77) can be used as a proxy for total PCDD/F exposure in this population and in populations with similar exposure profiles, but the correlation is lowest in cord tissue, where it may be necessary to measure an additional number of higher chlorinated dioxin-like PCBs to get a good proxy measure.

## Electronic supplementary material

Below is the link to the electronic supplementary material.

(PDF 119 KB)
