# Supplementary material for: Fetal exposure markers of dioxins and dioxin-like PCBs
Source: Environ Sci Pollut Res Int. 2018 Feb 15;25(12):11940–7. doi: 10.1007/s11356-018-1447-y (PMC5940721; doi:10.1007/s11356-018-1447-y)
Supplement: Supplementary file 1 — (PDF 119 KB) [file 11356_2018_1447_MOESM1_ESM.pdf]

Fetal Exposure Markers of Dioxins and Dioxin-like PCBs.

Supplementary Material

Erik Lampa, Akifumi Eguchi, Emiko Todaka and Chisato Mori

1 Descriptive statistics

1.1 Lipid base

Table S1: Descriptive statistics presented as Median (IQR). N is the number of observations above the LOD. xe$\pm y$ means $x \cdot 10^{\pm y}$. All units are pg/g-fat.

	Maternal blood		Cord blood		Cord tissue	
Contaminant	N	Median (IQR)	N	Median (IQR)	N	Median (IQR)
2,3,7,8-TeCDD	34	0.76 (0.66 – 0.95)	4	1.0 (0.96 – 1.0)	14	0.83 (0.73 – 1.1)
1,2,3,7,8-PeCDD	41	3.7 (2.7 – 4.3)	36	2.0 (1.6 – 2.5)	40	3.8 (2.9 – 5.0)
1,2,3,4,7,8-HxCDD	39	1.6 (1.1 – 2.0)	4	1.5 (1.4 – 1.7)	15	1.5 (1.2 – 1.6)
1,2,3,6,7,8-HxCDD	41	15 (11 – 18)	41	6.8 (5.6 – 8.0)	41	9.4 (6.7 – 11)
1,2,3,7,8,9-HxCDD	41	2.3 (1.8 – 3.0)	32	1.8 (1.4 – 2.1)	24	2.3 (1.7 – 3.0)
1,2,3,4,6,7,8-HpCDD	41	11 (8.7 – 16)	41	4.6 (3.5 – 6.8)	41	6.3 (5.2 – 8.5)
OCDD	41	53 (40 – 77)	41	14 (9.0 – 19)	41	17 (14 – 25)
2,3,7,8-TeCDF	40	0.78 (0.66 – 0.93)	24	0.73 (0.64 – 0.89)	15	0.78 (0.51 – 1.1)
1,2,3,7,8-PeCDF	21	0.67 (0.57 – 0.80)	5	0.93 (0.90 – 1.1)	6	0.68 (0.59 – 0.90)
2,3,4,7,8-PeCDF	41	10 (8.7 – 14)	41	5.0 (4.0 – 6.7)	41	10 (8.3 – 13)
1,2,3,4,7,8-HxCDF	41	2.5 (2.1 – 3.3)	39	1.4 (1.1 – 1.8)	40	2.7 (2.2 – 3.1)
1,2,3,6,7,8-HxCDF	41	3.3 (2.9 – 4.5)	41	2.3 (1.8 – 3.0)	41	3.1 (2.4 – 3.6)
1,2,3,7,8,9-HxCDF	0	–	0	–	0	–
2,3,4,6,7,8-HxCDF	39	1.4 (0.98 – 1.8)	16	1.1 (1.0 – 1.2)	29	1.2 (0.89 – 1.6)
1,2,3,4,6,7,8-HpCDF	41	2.3 (1.9 – 3.4)	39	1.7 (1.4 – 2.4)	39	2.4 (1.8 – 2.8)
1,2,3,4,7,8,9-HpCDF	0	–	0	–	0	–
OCDF	3	0.47 (0.45 – 0.48)	0	–	0	–
3,3',4,4'-TeCB(#77)	41	5.3 (4.2 – 6.6)	40	3.8 (3.1 – 5.2)	40	3.8 (3.1 – 5.3)
3,4,4',5-TeCB(#81)	41	0.37 (0.28 – 0.53)	18	0.31 (0.25 – 0.36)	33	0.43 (0.31 – 0.57)
2,3,3',4,4'-PeCB(#105)	41	3.3e+3 (2.4e+3 – 5.0e+3)	41	1.6e+3 (1.0e+3 – 2.3e+3)	41	2.5e+3 (2.0e+3 – 3.7e+3)
2,3,4,4',5-PeCB(#114)	41	4.0e+3 (3.2e+3 – 6.7e+3)	41	1.9e+3 (1.3e+3 – 3.0e+3)	41	3.1e+3 (2.6e+3 – 4.3e+3)
2,3',4,4',5-PeCB(#118)	41	1.6e+4 (1.0e+4 – 2.1e+4)	41	7.0e+3 (4.7e+3 – 1.1e+4)	41	1.2e+4 (8.0e+3 – 1.5e+4)
2',3,4,4',5-PeCB(#123)	41	2.3e+2 (1.7e+2 – 3.1e+2)	41	1.1e+2 (90 – 1.9e+2)	41	1.8e+2 (1.3e+2 – 2.4e+2)
3,3',4,4',5-PeCB(#126)	41	25 (18 – 33)	41	10 (7.6 – 15)	41	17 (13 – 23)
2,3,3',4,4',5-HxCB(#156)	41	2.7e+4 (1.9e+4 – 4.0e+4)	41	9.0e+3 (6.7e+3 – 1.5e+4)	41	1.5e+4 (1.3e+4 – 2-1e+4)
2,3,3',4,4',5'-HxCB(#157)	41	6.7e+3 (5.0e+3 – 1.0e+4)	41	2.7e+3 (2.0e+3 – 4.3e+3)	41	4.0e+3 (3.3e+3 – 5.7e+3)
2,3',4,4',5,5'-HxCB(#167)	41	2.0e+2 (1.5e+2 – 2.7e+2)	41	77 (60 – 1.2e+2)	41	1.2e+2 (87 – 1.6e+2)
3,3',4,4',5,5'-HxCB(#169)	41	7.3 (6.0 – 9.7)	41	2.5 (1.9 – 3.3)	41	4.0 (3.3 – 5.0)
2,3,3',4,4',5,5'-HpCB(#189)	41	6.0e+2 (4.7e+2 – 8.0e+2)	41	1.7e+2 (1.3e+2 – 2.5e+2)	41	2.7e+2 (2.2e+2 – 3.3e+2)

1.2 Wet weight

Table S2: Descriptive statistics presented as Median (IQR). N is the number of observations above the LOD. xe$\pm y$ means $x \cdot 10^{\pm y}$. All units are pg/g.

	Maternal blood		Cord blood		Cord tissue	
Contaminant	N	Median (IQR)	N	Median (IQR)	N	Median (IQR)
2,3,7,8-TeCDD	34	4.7e-03 (3.7e-03 – 5.9e-03)	4	2.2e-03 (2.0e-03 – 2.5e-03)	14	1.1e-03 (1.0e-03 – 1.6e-03)
1,2,3,7,8-PeCDD	41	2.2e-02 (1.5e-02 – 2.6e-02)	36	4.8e-03 (4.0e-03 – 6.1e-03)	40	4.8e-03 (3.5e-03 – 6.1e-03)
1,2,3,4,7,8-HxCDD	39	9.5e-03 (6.6e-03 – 1.2e-02)	4	3.4e-03 (2.9e-03 – 3.8e-03)	15	2.0e-03 (1.8e-03 – 2.2e-03)
1,2,3,6,7,8-HxCDD	41	7.7e-02 (6.3e-02 – 1.1e-01)	41	1.7e-02 (1.4e-02 – 2.1e-02)	41	1.1e-02 (8.9e-03 – 1.4e-02)
1,2,3,7,8,9-HxCDD	41	1.3e-02 (1.0e-02 – 1.9e-02)	32	4.1e-03 (3.2e-03 – 5.3e-03)	24	2.8e-03 (1.8e-03 – 3.8e-03)
1,2,3,4,6,7,8-HpCDD	41	6.0e-02 (5.0e-02 – 7.9e-02)	41	1.1e-02 (9.7e-03 – 1.6e-02)	41	7.7e-03 (6.2e-03 – 1.0e-02)
OCDD	41	2.8e-01 (2.1e-01 – 4.3e-01)	41	3.3e-02 (2.4e-02 – 4.7e-02)	41	2.0e-02 (1.6e-02 – 3.1e-02)
2,3,7,8-TeCDF	40	4.6e-03 (3.6e-03 – 5.2e-03)	24	1.8e-03 (1.7e-03 – 1.9e-03)	15	9.9e-04 (6.0e-04 – 1.1e-03)
1,2,3,7,8-PeCDF	21	4.3e-03 (3.2e-03 – 5.3e-03)	5	2.5e-03 (2.2e-03 – 2.5e-03)	6	9.3e-04 (8.7e-04 – 1.0e-03)
2,3,4,7,8-PeCDF	41	6.0e-02 (4.7e-02 – 7.7e-02)	41	1.3e-02 (1.0e-02 – 1.7e-02)	41	1.3e-02 (9.7e-03 – 1.6e-02)
1,2,3,4,7,8-HxCDF	41	1.4e-02 (1.0e-02 – 1.8e-02)	39	3.6e-03 (2.9e-03 – 4.3e-03)	40	3.3e-03 (2.4e-03 – 3.8e-03)
1,2,3,6,7,8-HxCDF	41	1.9e-02 (1.6e-02 – 2.5e-02)	41	5.8e-03 (4.9e-03 – 7.2e-03)	41	3.7e-03 (3.0e-03 – 4.4e-03)
1,2,3,7,8,9-HxCDF	0	–	0	–	0	–
2,3,4,6,7,8-HxCDF	39	8.0e-03 (5.7e-03 – 9.7e-03)	16	2.6e-03 (2.2e-03 – 2.8e-03)	29	1.6e-03 (1.1e-03 – 1.8e-03)
1,2,3,4,6,7,8-HpCDF	41	1.4e-02 (1.0e-02 – 2.0e-02)	39	4.3e-03 (3.5e-03 – 5.3e-03)	39	2.6e-03 (2.2e-03 – 3.5e-03)
1,2,3,4,7,8,9-HpCDF	0	–	0	–	0	–
OCDF	3	3.1e-03 (2.8e-03 – 3.1e-03)	0	–	0	–
3,3',4,4'-TeCB(#77)	41	2.9e-02 (2.5e-02 – 3.5e-02)	40	9.2e-03 (7.5e-03 – 1.2e-02)	40	4.7e-03 (3.3e-03 – 6.2e-03)
3,4,4',5-TeCB(#81)	41	2.1e-03 (1.6e-03 – 3.0e-03)	18	7.3e-04 (6.3e-04 – 8.6e-04)	33	5.3e-04 (4.0e-04 – 6.3e-04)
2,3,3',4,4'-PeCB(#105)	41	19 (13 – 30)	41	4.0 (2.8 – 5.0)	41	3.0 (2.1 – 4.7)
2,3,4,4',5-PeCB(#114)	41	27 (19 – 33)	41	5.0 (3.3 – 7.0)	41	4.3 (2.7 – 5.0)
2,3',4,4',5-PeCB(#118)	41	97 (63 – 1.2e+02)	41	19 (13 – 24)	41	13 (9.0 – 20)
2',3,4,4',5-PeCB(#123)	41	1.4 (0.90 – 2.1)	41	0.29 (0.23 – 0.40)	41	0.22 (0.14 – 0.31)
3,3',4,4',5-PeCB(#126)	41	0.16 (9.3e-02 – 0.20)	41	2.6e-02 (2.1e-02 – 3.5e-02)	41	2.1e-02 (1.3e-02 – 3.1e-02)
2,3,3',4,4',5-HxCB(#156)	41	1.7e+02 (1.1e+02 – 2.0e+02)	41	22 (17 – 32)	41	20 (14 – 26)
2,3,3',4,4',5'-HxCB(#157)	41	43 (30 – 53)	41	6.7 (5.3 – 10)	41	5.7 (4.0 – 7.0)
2,3',4,4',5,5'-HxCB(#167)	41	1.3 (0.90 – 1.5)	41	0.20 (0.16 – 0.28)	41	0.14 (0.11 – 0.20)
3,3',4,4',5,5'-HxCB(#169)	41	4.3e-02 (3.2e-02 – 6.0e-02)	41	6.0e-03 (5.3e-03 – 8.7e-03)	41	5.0e-03 (4.0e-03 – 6.7e-03)
2,3,3',4,4',5,5'-HpCB(#189)	41	3.7 (2.5 – 5.0)	41	0.43 (0.32 – 0.63)	41	0.37 (0.24 – 0.43)
